# Do Young Chinese Children Gain Anthropomorphism after Exposure to Personified Touch-Screen and Board Games?

**DOI:** 10.3389/fpsyg.2017.00055

**Published:** 2017-01-25

**Authors:** Hui Li, Yeh Hsueh, Fuxing Wang, Xuejun Bai, Tao Liu, Li Zhou

**Affiliations:** ^1^Academy of Psychology and Behavior, Tianjin Normal UniversityTianjin, China; ^2^College of Education, University of Memphis, MemphisTN, USA; ^3^School of Psychology, Central China Normal UniversityWuhan, China; ^4^School of Management, Zhejiang UniversityHangzhou, China

**Keywords:** touch screen, game, transfer, children, anthropomorphism

## Abstract

Research shows that preschoolers are likely to anthropomorphize not only animals, but also inanimate toy after being exposed to books that personify these objects. Can such an effect also arise through young children’s use of touch-screen games? The present study is the first to examine whether playing a touch-screen personified train game affects young children’s anthropomorphism of real trains. Seventy-nine 4- and 6-year-old children were randomly assigned to play either a touch-screen game or a board game of Thomas the Tank Engine for 10 min. They completed the Individual Differences in Anthropomorphism Questionnaire–Child Form (IDAQ-CF) (two subscales: Technology/Inanimate Nature, Animate Nature) and an additional four items about the anthropomorphism of real trains, before (T1) and after (T2) the game. Overall results showed that children manifested a small but statistically significant increase in anthropomorphizing of real trains after their exposure to both games, claiming that real trains were like humans. Interestingly, 4-year-old children in the board game group tended to anthropomorphize real trains more than those in the touch-screen group, whereas the reverse was true for the 6-year-old children. The results suggest that touch-screen games may delay the decline of children’s anthropomorphism during the cognitive and socio-emotional transition that occurs in children aged 5–7. These findings have implications for future research on how touch-screen games increase children’s anthropomorphism of the real world, and more generally, for evaluation of the influence of the growing use of touch-screen games on young children’s learning.

## Introduction

Recent survey data suggest that young children start to use tablets at a very early age, most often to watch videos and play video games (Common Sense Media, 2013). This playful use of various media affects their development and learning (Moreno, 2016). A great amount of anthropomorphism exists in interactive games designed for young children, with inanimate objects and animals being made to look and act like humans (for a review, see Hartman and Vorderer, 2009), a phenomenon already widely observed in children’s picture books about animals and their natural environment (Marriott, 2002).

In recent years, touch-screen devices have become prevalent in children’s lives (Cristia and Seidl, 2015), being more interactive than earlier play media such as picture books and board games. In this area of research, three features of touch-screen devices stand out: interactivity, tailorability, and progression (Christakis, 2014). The salient feature of interactivity of touch screens allows children not only to anthropomorphize the character in a game, but also to get reactions by touching it. Such multimodal stimulation creates a sense of presence (Preston, 2007) in which the user feels as though he or she were physically present in the scene (Benski and Fisher, 2013).

But recent research in spatial cognition points to another critical feature of the touch screen: it is 2-dimensional (2D). Young children’s understandings of 2-dimensional representations improve significantly between 5 and 6 (Frick and Newcombe, 2015; Lytle et al., 2015). Studies showed that although 5-year-olds performed well on a task involving a 2D image rotating on a touch-screen, 4-year-olds performed only at chance (Frick et al., 2013a,b). In other words, children older than 5 become able to understand and manipulate 2D representations.

Then, does young children’s exposure to a personified object in 2D touch-screen games or 3D board games affect their anthropomorphic understanding of the world? One recent study suggested that preschoolers’ exposure to an anthropomorphic storybook increased the likelihood that they would view real trains as possessing human qualities (Li et al., 2015). Could the effect of anthropomorphism found in storybooks also exist in children’s exposure to touch-screen games? As touch-screen devices become increasingly accessible to young children, their impact on children’s anthropomorphism raises important but under-researched questions about young children’s thinking and behavior. We designed two similar games, one board game and one touch-screen game, with an anthropomorphic character, Thomas the Tank Engine, to investigate the effects of playing each game on young children’s anthropomorphism of trains in the real world. Thomas was chosen for this study because it is a popular personified train character, and it is easy for preschoolers to relate to a real train.

Anthropomorphism has been defined as attributing uniquely human characteristics to non-human agents or events, like animals and vehicles (Waytz et al., 2010). Anthropology and psychology have seen a long-lasting interest in anthropomorphism and the related concept of animism (e.g., Tylor, 1871; McDougall, 1911; Piaget, 1929; Looft and Bartz, 1969; Epley et al., 2007). Previous research has shown that 4- and 5-year-old urban children describe their biological knowledge of animals in an anthropocentric way (Springer and Keil, 1989; Waxman and Medin, 2007). Some argue that the anthropomorphism of animals can hinder children’s understanding of the biological world (Ganea et al., 2011, 2014; Legare et al., 2013). Like animism, which declines with age (Jahoda, 1958; Inagaki, 1989), anthropomorphism declines noticeably from early to middle childhood (Severson and Lemm, 2016) following the so-called period of “the age of reason” (White, 1996).

We designed the present study to assess the effect of a touch-screen game and a board game on 4- and 6-year-old children’s anthropomorphism. Each age group was divided into two game groups, namely, board game and touch-screen game. The game was organized around the activities of Thomas the Tank Engine, a personified train. Children’s general anthropomorphism and specific anthropomorphism of trains were assessed before the game and 1 day later. Our main question was: Would an animated touch-screen game affect young children’s anthropomorphism in a similar way as an animated board game would? For the reasons above, our first hypothesis was that children would show increased anthropomorphism in the touch-screen condition, just as they would in the board game condition. Our second hypothesis was that the older children were, the less they would anthropomorphize real trains.

The focus of this study was on the changes from T1 to T2, and interactions between this change and other factors, rather than a direct comparison between the two types of games. As noted, we expected the pattern of results to be similar across the two types of games. In cases where we did find differences between the two media, we offer tentative interpretations, as we did not make hypotheses about these differences.

## Materials and Methods

### Participants

Seventy-nine children from a kindergarten in central China participated in the study, 39 4-year-olds (16 girls, *M*_age_ = 54.97 months, *SD* = 2.62) and 40 6-year-olds (15 girls, *M*_age_ = 73.85 months, *SD* = 1.46). They were randomly assigned to two groups: board game and touch-screen game. Parents reported that the children first saw a real train between their first and second birthday, both in the touch-screen group (*M*_age_ = 15.9 months, *SD* = 13.9) and in the board group (*M*_age_ = 22.7 months, *SD* = 11.1). The first encounter with Thomas the Tank Engine in different media was similar between the two groups (**Table 1**). There was no significant difference in their usage frequency, nor was there any significant group difference in children’s prior knowledge of Thomas the Tank Engine in other media (**Table 2**). Additionally, during the 2 weeks before the study, the two groups did not differ in their exposure to Thomas the Tank Engine either on iPad or in board games.

**Table 1:** Children’s age (months) at first encounter with Thomas the Tank Engine in different media.

Types	Storybooks			DVD/Television			Board game			Touch-screen game		
	*N*	*M*	*SD*	*N*	*M*	*SD*	*N*	*M*	*SD*	*N*	*M*	*SD*
Board	27	27.5	7.40	27	24.0	11.53	23	27.8	11.25	5	41.4	14.76
Touch-screen	28	29.5	11.55	24	28.9	9.99	28	28.1	11.48	6	32.5	12.32

**Table 2:** Children’s basic anthropomorphism at T1 and T2 in 4-year-olds and 6-year-olds.

Age	Group	*N*	T1TIN		T1ANI		T2TIN		T2 ANI	
			*M*	*SD*	*M*	*SD*	*M*	*SD*	*M*	*SD*
4	Board	19	1.06	0.88	1.44	0.89	1.25	0.90	1.34	0.88
	Touch-screen	20	0.88	0.71	1.44	0.70	0.92	0.71	1.04	0.61
6	Board	19	0.75	0.76	1.57	0.74	0.91	0.82	1.26	0.74
	Touch-screen	21	0.64	0.57	1.54	0.56	0.69	0.74	1.26	0.58

*T1, Time 1; T2, Time 2; TIN, Technology/Inanimate-Nature; ANI, Animate Nature.*

Parents and teachers provided consent for all the participating children. Each child was given a sticker as a token of appreciation at the end of participation. The present research was approved by the Committee on Ethical Research Practice of the university with which the third author is affiliated.

### Measures

Individual Differences in Anthropomorphism Questionnaire-Child Form (IDAQ-CF), a highly reliable measure (12 items, α = 0.80) (Severson and Lemm, 2016), was translated into Chinese and then back-translated into English to ensure the original meanings. The original IDAQ-CF contained three subscales: Technological Nature with four items: robot, TV, car, and computer; Inanimate Nature with four items: mountain, ocean, tree, wind; and Animate Nature with four items: cheetah, turtle, insect, and lizard. However, because the Technological and Inanimate Nature items loaded well onto the same factor (Severson and Lemm, 2016), they were combined in our analyses, resulting in two subscales: Technology/Inanimate-Nature (mean score for items 1–8), and Animate Nature (mean score for items 9–12). The same 12-item Chinese version of IDAQ-CF was used to measure the level of anthropomorphism at Times 1 and 2.

Each item had two questions for the child to answer. First, for example, does a TV have feelings, like happy and sad? If the child said no or gave no response, the child completed the item with a score of 0. If yes, the child was asked the second question, how much? On completing the second question, the child received a score between 1 and 3 to indicate a little, or a medium amount, or a lot. An average score across all items fell within the range of 0–3. This scoring method also applied to four new questions added to measure the anthropomorphism of real trains as follows.

Q13. Does a real train have feelings, like being happy and sad? If yes, how much feeling does a real train have?
Q14. Does a real train know what it is? If yes, how much does a real train know what it is?
Q15. Does a real train do things on purpose? If yes, how much does a real train do things on purpose?
Q16. Does a real train think for itself? If yes, how much does a real train think for itself?

At both T1 and T2, all 16 items were presented randomly. Before the 16 items were presented at T1and T2, three pairs of training questions from the IDAQ-CF were administered to ensure that children understood the type of questions that would be asked and how to respond to them. For example, “Do you like candy (broccoli, carrots)? If yes, how much?” The same scoring system was used as for the above 16 items. The scores of training questions were not included in the data analysis because they were only used for children’s familiarity with the scale.

Each child’s play session was video recorded to examine how engaged each child was in playing. Later, the video time code was used in the unit of seconds to mark the durations of the child’s eyes-on-the-game behaviors and the child’s eyes-off-the-game behaviors. The ratio of the total duration of eyes-on-the-game (attention) to the total time of playing was used as an index of the child’s play engagement. One rater coded all the videos during children’s play. The other rater coded 10 randomly selected videos (five from each condition). The inter-rater reliability for the index of attention was *r* = 0.997, *p* < 0.01 and for the index of distraction, *r* = 0.937, *p* < 0.01.

A parent questionnaire was used to assess the children’s first encounter and life experience with real trains, and how often the children were exposed to the board game and touch-screen game of Thomas the Tank Engine during the 2 weeks before the study. No differences were found between age groups or between the two game groups in terms of previous game experience, first encounter or real experience with real trains or Thomas the Tank Engine (*p*s > 0.05).

### Materials and Procedure

Both games focused on playing with the personified characteristics of Thomas the Tank Engine. The children were asked to help Thomas transport people and cargo to prepare for a birthday party by moving (board game) or touching (touch-screen game on iPad) Thomas. The two games were designed to be similar to each other, using Thomas the Tank Engine as the personified character and having it follow a similar routine. Both groups were asked to add a cave over the railroad and plant a tree by the railroad. They also needed to transport goods represented by stickers including the following images: orange, papaya, pear, cherry, and lemon, a bunch of balloons, a group of seven children, Santa Claus with a gift box.

The study went on for 2 days. On Day 1, every child completed the pre-test using the IDAQ-CF. On Day 2, they played the assigned game for 10 min and completed the post-test. One experimenter administered the play session, but another conducted both pre- and post-test in order to avoid the experimenter effect. Before playing the game, the child was told, “You will play a game to help Thomas transport cargo and people today. Look! This is our friend Thomas. He is very nice, and willing to help others. He knows he is a train, and he often helps his friends. There will be a birthday party today. Can you help Thomas finish the task?” Then the experimenter showed the child where to start and to end on the railroad, and how to move the train. The child was also asked to put the tree and the cave along the railroad. After the experimenter verified the child’s understanding of the game, the child began playing independently, sometimes with encouragement.

## Results

### Basic Anthropomorphic Effects

A 2 (age) × 2 (condition) × 2 (time) mixed-effects ANOVA was conducted, with age (4, 6) and condition (board game, touch-screen game) as between-subjects factors and time (T1, T2) as a within-subjects factor. The two dependent variables were the two key score categories of the IDAQ-CF: Technology/Inanimate-Nature, Animate-Nature.

For the Technology/Inanimate-Nature score, there was a main effect of time: the children showed marginally higher anthropomorphic scores at T2 (*M* = 0.94, *SE* = 0.09) than at T1 (*M* = 0.83, *SE* = 0.08), *F*(1,75) = 4.00, *p* = 0.049, $\eta_{p}^{2}$ = 0.05. The main effect of age [*F*(1,75) = 2.91, *p* = 0.09, $\eta_{p}^{2}$ = 0.04] and condition [*F*(1,75) = 0.83, *p* = 0.36, $\eta_{p}^{2}$ = 0.01], and the interactions of time and condition [*F*(1,75) = 0.83, *p* = 0.37, $\eta_{p}^{2}$ = 0.01], time and age [*F*(1,75) = 0.01, *p* = 0.92, $\eta_{p}^{2}$ < 0.001], and condition and age [*F*(1,75) = 0.12, *p* = 0.73, $\eta_{p}^{2}$ = 0.002], as well as the three-way interaction of age and condition and time [*F*(1,75) = 0.02, *p* = 0.90, $\eta_{p}^{2}$ < 0.001] were all non-significant.

For the Animate-Nature score, there was also a main effect of time: however, contrary to expectations, the children showed significantly lower anthropomorphic scores at T2 (*M* = 1.22, *SE* = 0.08) than at T1 (*M* = 1.42, *SE* = 0.08), *F*(1,75) = 9.44, *p* = 0.003, $\eta_{p}^{2}$ = 0.11. The main effect of age [*F*(1,75) = 1.50, *p* = 0.22, $\eta_{p}^{2}$ = 0.020] and condition [*F*(1,75) = 0.95, *p* = 0.33, $\eta_{p}^{2}$ = 0.013], and the interaction of time and condition [*F*(1,75) = 0.01, *p* = 0.94, $\eta_{p}^{2}$ < 0.001], time and age [*F*(1,75) = 2.75, *p* = 0.10, $\eta_{p}^{2}$ = 0.035], and condition and age [*F*(1,75) = 0.31, *p* = 0.58, $\eta_{p}^{2}$ = 0.004], as well as the three-way interaction of age and condition and time [*F*(1,75) = 0.01, *p* = 0.91, $\eta_{p}^{2}$ < 0.001] were all non-significant.

### Anthropomorphic Scores for Real Trains

A 2 (age) × 2 (condition) × 2 (time) mixed-effects ANOVA was conducted to compare the anthropomorphic scores for real trains, with age (4, 6) and condition (board game, touch-screen game) as between-subjects factors and time (T1, T2) as a within-subjects factor. The dependent variable was the score for anthropomorphism about trains. Results showed that the main effect of time was significant [*F*(1,75) = 5.54, *p* = 0.02, $\eta_{p}^{2}$ = 0.069], in that the anthropomorphic scores on pretest (*M* = 0.90, *SE* = 0.09) were lower than those on post-test (*M* = 1.05, *SE* = 0.11). Also, the main effect of age was significant, *F*(1,75) = 4.99, *p* = 0.03, $\eta_{p}^{2}$ = 0.062, in that 4-year-old children had higher scores (*M* = 1.19, *SE* = 0.14) than 6-year-old children (*M* = 0.75, *SE* = 0.14).

**FIGURE 1:**
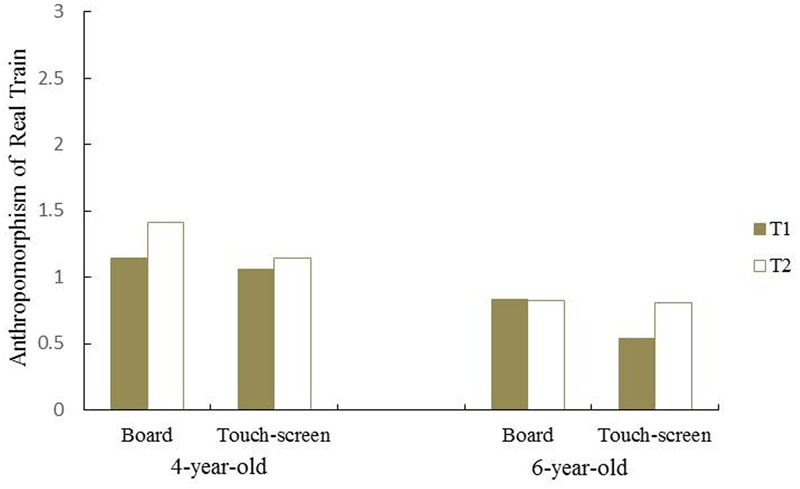
**Pre and Post Anthropomorphism of real train at T1 and T2 in 4-year-olds and 6-year-olds**.

The three two-way interactions, namely time and age [*F*(1,75) = 0.10, *p* = 0.76, $\eta_{p}^{2}$ = 0.001], time and condition [*F*(1,75) = 0.16, *p* = 0.69, $\eta_{p}^{2}$ = 0.002], and age and condition [*F*(1,75) = 0.003, *p* = 0.96, $\eta_{p}^{2}$ < 0.001] were non-significant. Interestingly, however, there was a trend for a three-way interaction among age, condition, and time, [*F*(1,75) = 3.37, *p* = 0.07, $\eta_{p}^{2}$ = 0.04]. A simple effects analysis revealed that the 4-year-olds in the board game condition anthropomorphized real trains in T2 (*M* = 1.41, *SE* = 0.22) more than T1 (*M* = 1.15, *SE* = 0.19) [*F*(1,75) = 4.24, *p* = 0.04, $\eta_{p}^{2}$ = 0.054]. However, the 6-year-olds in the touch-screen condition anthropomorphized real trains in T2 (*M* = 0.81, *SE* = 0.22) more than T1 (*M* = 0.54, *SE* = 0.19) [*F*(1,75) = 4.66, *p* = 0.03, $\eta_{p}^{2}$ = 0.058] (**Figure 1**). In short, at T2, 4-year-old children in the board game condition gained more anthropomorphism of real trains whereas 6-year-old children did so in the touch-screen condition.

### Children’s Attention during Play

In order to understand how children’s engagement during play might affect the results, two raters coded the time of attention (i.e., total time attending divided by total play time) and the time of distraction (i.e., total time distracted divided by total play time) separately to create the indexes of attention and distraction, using the time code on the video. After setting aside two outliers which were three standard deviations above or below the children’s mean time of attention, a 2 (age) × 2 (condition) MANOVA was conducted to test whether there were differences in the ratio of attention and distraction.

Results showed main effects of age [Wilk’s Λ = 0.941, *F*(1,73) = 4.57, *p* = 0.036, $\eta_{p}^{2}$ = 0.059] and condition [Wilk’s Λ = 0.945, *F*(1,73) = 4.27, *p* = 0.042, $\eta_{p}^{2}$ = 0.055]. Follow-up analyses showed that there were significant age differences in the ratio of attention, in that 6-year-old children (*M* = 0.99, *SE* = 0.004) had higher attention ratio than 4-year-old children (*M* = 0.97, *SE* = 0.006), *F*(1,73) = 4.57, *p* = 0.036, $\eta_{p}^{2}$ = 0.059; however, 4-year-old children (*M* = 0.03, *SE* = 0.06) had a higher ratio of distraction than 6-year-old children (*M* = 0.01, *SE* = 0.004), *F*(1,73) = 4.57, *p* = 0.036, $\eta_{p}^{2}$ = 0.059. There were also condition differences in the ratio of attention, in that children in the touch-screen game condition had a higher attention ratio (*M* = 0.99, *SE* = 0.002) than those in the board game condition (*M* = 0.97, *SE* = 0.006), *F*(1,73) = 4.27, *p* = 0.042, $\eta_{p}^{2}$ = 0.055; in contrast, children in the board game condition had a higher distraction ratio (*M* = 0.03, *SE* = 0.04) than those in the touch-screen game condition (*M* = 0.01, *SE* = 0.002), *F*(1,73) = 4.27, *p* = 0.042, $\eta_{p}^{2}$ = 0.055.

A series of 2 (age) × 2 (condition) × 2 (time) mixed-effects ANCOVAs, ratio of attention as covariate, IDAQ-CF scores and trains scores as dependent variables, were conducted to test whether ratio of attention influenced the result. Results showed that the effect of the covariate was non-significant, *F*s (1,72) < 3.84, *p*s > 0.05.

## Discussion

The present study tested whether Chinese young children would show greater anthropomorphism about real trains after being exposed to a board game or a touch-screen game that personified Thomas the Tank Engine. We assessed children’s anthropomorphism of real trains before and after playing one of the two games, both of which featured the personified train that has a human face on the front. It is important to note that there was an overall increase in children’s anthropomorphism of real trains following either game, which replicates other researchers’ findings (e.g., Herrmann et al., 2010; Ganea et al., 2014; Li et al., 2015) and supports the view that young children’s exposure to media with strong personified features can increase their anthropomorphism in the real world. The results support our first hypothesis that children would be likely to anthropomorphize real trains after playing either game. However, they only partially support our second hypothesis that the older children were, the less they would anthropomorphize real trains; specifically, the 3D board game data support this hypothesis but the 2D touch screen game data do not.

In our sample, there was a non-significant age difference in children’s general anthropomorphism (assessed by the two IDAQ-CF subscales) both before and after the game. Such a non-significant age difference in anthropomorphism of Technology/Inanimate-Nature subscale is consistent with the data the IDAQ designers obtained on the same subscale (Severson and Lemm, 2016). Although they found a significant age difference between 5 and 9 in the Animate-Nature subscale, this subscale was not particularly sensitive to the two younger age groups, 4 and 6. It is not surprising that our finding shows no significant age difference because participants in the present study were even younger.

It is interesting to note that Animate-Nature scores decreased from T1 to T2, in both age groups, but Technology/Inanimate-Nature scores did not. This finding is consistent with a study by Severson and Lemm (2016), who found that children who endorsed anthropomorphism of animals were less likely to ascribe animate characteristics to the robot and that the two IDAQ-CF subscales might have some inverse relationship. One possible interpretation of this result is that children were exposed to animate items only through verbal and visual representations while they were physically and mentally engaged with a personified train in the game. Because children did not have difficulty identifying living things as animate by correlating various cues (Arterberry and Bornstein, 2002; Rakison and Lupyan, 2008), their anthropomorphism of animals might remain stable without the game conditions. In the current study, the game conditions did not direct children’s attention to these living things further, but provided more dynamic cues, namely, human-typical acts, for children to process. These dynamic cues involved agency (e.g., making the train do things), intentionality and goal-directedness (e.g., transporting people and planting trees) (for a review, see Opfer and Gelman, 2011). The child’s mind processed the immediate cognitive input from the personified acts of Thomas while the mind might leave little room for processing the information from the Animate subscale. A strongly personified Thomas the Tank Engine made the animate less important in the child’s mind than before the game, resulting in a small but significant decrease.

However, it should be noted that the two game conditions in the present study highlighted the personified feature of the object, Thomas the Tank Engine, and the interactive feature of the game. These two features were not integral to the IDAQ-CF, which is a verbal measure. Adding the four train items to the IDAQ-CF was driven by our research question about the potential impact of the two game conditions on young children’s anthropomorphism of real trains. It becomes clear that this apparent inconsistency above warrants further research to assess the possible difference in young children’s anthropomorphism between verbal exposure and physically involved exposure to a medium, including exposure to the realistic train.

There was a trend for an interesting interaction between age group and game type in terms of anthropomorphizing real trains. The 4-year-old group showed more anthropomorphism of real trains after playing the board game, whereas for the 6-year-old group the effect was larger after playing the touch-screen game. As mentioned, there is reason to believe that the age difference in children’s anthropomorphism of real trains may be moderated by the game type, especially due to the game dimensionality. Recent research shows that 4-year-olds are able manually and observationally respond to 3D objects correctly, but respond to 2D objects at chance in all conditions. However, there is a developmental watershed between ages 5 and 6. Six-year-olds respond significantly better to the 2D objects (Frick et al., 2013a,b; Frick and Newcombe, 2015; Lytle et al., 2015).

These findings help explain why 4-year-olds showed a higher level of anthropomorphism toward the real trains at T2 than 6-year-olds in the board game group: 3D objects are easier or more meaningful, and therefore, they were more susceptible to the influence of the personified train. In contrast, although 6-year-olds developmentally were less likely to anthropomorphize inanimate objects, the touch-screen game appeared to set back this developmental progress at T2 with the personified 2D object, Thomas the Tank Engine, probably because the 2D image manipulation is an emerging ability. Other researchers note that 6-year-olds’s performance on 2D images are far from perfect and even 7-year-olds only reach 79% accuracy (Frick and Newcombe, 2015). By the same logic, we may see that 4-year-olds in the touch screen condition and the 6-year-olds in the board game condition did not show significant change from T1 to T2, but for different reasons.

Sociocultural researchers also show evidence that children at six experience noticeable cognitive and socio-emotional transitions (Rogoff et al., 1975; White, 1996), and their tendency to anthropomorphize actual objects should have decreased unless the media environment, the touch-screen game in this case, interferes with this transition. As newly fashioned cultural tools, touch-screen media with its immediately interactive engagement with 2-dimensional objects may present parents, teachers and psychologists a new set of developmental questions to pursue in both practice and research. It leads us to speculate that personified media in general, including the fast-growing market of tablet game apps, can have similar effects on young children.

This interpretation echoes the concern that science educators of young children have raised. Recall the earlier argument that the anthropomorphized animals in children’s literature can hinder children’s understanding of the biological world (Gallant, 1981; Legare et al., 2013; Ganea et al., 2014). Some insist that it would be better to depict the world in children’s books realistically rather than anthropomorphically (Richert et al., 2009; Ganea et al., 2011). The possible touch-screen-facilitated increase of anthropomorphism in 6-year-old Chinese children may provide the first evidence for science educators to further consider the possible effect of tablets in teaching biological science to young children.

It is interesting to note that 6-year-olds’ attention ratio was on average greater than 4-year-olds’. This finding is in line with the observation of attention span in child development. As children grow into middle childhood, they have more deliberate and self-regulated control of attention (Dossett and Burns, 2000). However, what is more interesting is that children’s engagement in the touch-screen condition is greater than that in the board game condition. Taking these differences together with the interaction trend reported above, we may ask whether such higher level of engagement with the touch-screen game would be a reason for intensifying 6-year-olds’ tendency to anthropomorphize real trains. This is a question for our future research.

The current research used a between-subjects design, a design observable in similar studies in two conditions (touch-screen or electronic toy version and physical toy version) without a control group (e.g., Zosh et al., 2015; Huber et al., 2016) because the focus of the study was on the changes from T1 to T2, and interactions between this change and other factors, rather than a direct comparison between the two types of games. In fact, we expected that children’s anthropomorphism would increase from pretest to post-test, following a similar pattern across the two types of games. However, future research can include a control condition to address the effect of different media treatments.

In summary, the present research suggests that Chinese 4- and 6-year-old children can show greater anthropomorphism about a certain object (in this case, a train) by playing personified games involving the object in a certain context. The board game context had a greater effect on 4-year-olds due to its 3-dimensional quality while the touch screen game context affected 6-year-olds paradoxically due to their developmental gain in spatial cognition. Our study offers tentative evidence for understanding a new dimension of anthropomorphism in young children. Media play an important role in representing an anthropomorphic world to children, and touch-screen games might contribute more to 6-year-old children’s anthropomorphism than board games. This first attempt to examine the role touch-screen media play in young children’s anthropomorphism provides new directions for our future research.

## Ethics Statement

This study was approved by the Institutional Review Board of Central China Normal University. All the parents had signed the consent inform before the study.

## Author Contributions

HL developed the study concept. All authors contributed to the study design. HL, FW, and LZ performed the experiment, and FW conducted the statistical analyses. HL and YH were primarily responsible for writing the manuscript, with all remaining authors providing critical revisions. All authors approved the final version of the manuscript for submission.

## Conflict of Interest Statement

The authors declare that the research was conducted in the absence of any commercial or financial relationships that could be construed as a potential conflict of interest.
